# Administration of indwelling urinary catheters in four Australian Hospitals: cost-effectiveness analysis of a multifaceted nurse-led intervention

**DOI:** 10.1186/s12913-021-06871-w

**Published:** 2021-08-31

**Authors:** Rod Ling, Michelle Giles, Andrew Searles

**Affiliations:** 1 Hunter Medical Research Institute, New Lambton Heights, NSW Australia; 2 University of Newcastle, School of Medicine and Public Health, Callaghan, NSW 2308 Australia; 3 Hunter New England Local Health District, Nursing and Midwifery Centre, Newcastle, NSW Australia

**Keywords:** Catheterisation, Costs and costs analysis, Nurses, Urinary Tract Infections

## Abstract

**Background:**

Urinary catheters are useful among hospital patients for allowing urinary flows and preparing patients for surgery. However, urinary infections associated with catheters cause significant patient discomfort and burden hospital resources. A nurse led intervention aiming to reduce inpatient catheterisation rates was recently trialled among adult overnight patients in four New South Wales hospitals. It included: ‘train-the trainer’ workshops, site champions, compliance audits and promotional materials. This study is the ‘in-trial’ cost-effectiveness analysis, conducted from the perspective of the New South Wales Ministry of Health.

**Methods:**

The primary outcome variable was catheterisation rates. Catheterisation and procedure/treatment data were collected in three point prevalence patient surveys: pre-intervention (*n* = 1630), 4-months (*n* = 1677), and 9-months post-intervention (*n* = 1551). Intervention costs were based on trial records while labour costs were gathered from wage awards. Incremental cost effectiveness ratios were calculated for 4- and 9-months post-intervention and tested with non-parametric bootstrapping. Sensitivity scenarios recalculated results after adjusting costs and parameters.

**Results:**

The trial found reductions in catheterisations across the four hospitals between preintervention (12.0 % (10.4 − 13.5 %), *n* = 195) and the 4- (9.9 % (8.5 − 11.3 %), *n* = 166 ) and 9- months (10.2 % (8.7 − 11.7 %) *n* = 158) post-intervention points. The trend was statistically non-significant (*p* = 0.1). Only one diagnosed CAUTI case was observed across the surveys. However, statistically and clinically significant decreases in catheterisation rates occurred for medical and critical care wards, and among female patients and short-term catheterisations. Incremental cost effectiveness ratios at 4-months and 9-months post-intervention were $188 and $264. Bootstrapping found reductions in catheterisations at positive costs over at least 72 % of iterations. Sensitivity scenarios showed that cost effectiveness was most responsive to changes in catheterisation rates.

**Conclusions:**

Analysis showed that the association between the intervention and changes in catheterisation rates was not statistically significant. However, the intervention resulted in statistically significant reductions for subgroups including among short-term catheterisations and female patients. Cost-effectiveness analysis showed that reductions in catheterisations were most likely achieved at positive cost.

**Trial Registration:**

Registered with the Australian New Zealand Clinical Trials Registry (ACTRN12617000090314). First hospital enrolment, 15/11/2016; last hospital enrolment, 8/12/2016.

**Supplementary Information:**

The online version contains supplementary material available at 10.1186/s12913-021-06871-w.

Full list of author information available at end of article.

## Background

Hospital-associated urinary tract infections (HAUTIs) are a significant hospital resource issue in Australia, causing the use of 380,600 extra public hospital bed-days among infected patients each year [1]. Among HAUTIs, it is estimated that catheter acquired urinary tract infections (CAUTIs) make up 70–80 % [2, 3]. CAUTI rates may be seen in the context of hospital nursing practice[4, 5] being related to appropriateness of catheter insertions and timeliness of removals [6]. These issues can be addressed through ‘bundles’, or collections of evidence-based nursing practices [6, 7]. Such a set of practices is the subject of a pre-post trial study - No-CAUTI - conducted in four hospitals in the Australian state of New South Wales (NSW) in 2018-19 [8–11]. This nurse-led intervention aimed to (1) reduce unnecessary urinary catheterisation procedures; and (2) increase adherence to evidence-based guidelines for catheter care and consideration of when urinary catheterisations are warranted [9–11]. Both aspects of care can potentially reduce CAUTI, along with the associated and consequential CAUTI-related treatment costs. The aim of this present study is to identify the ‘within-trial’ cost-effectiveness of the No-CAUTI intervention from the cost perspective of the state public sector health authority, the NSW Ministry of Health. Analysis was conducted and presented for a decision-making setting.

## Literature Review

A brief review was undertaken to identify the methods used in similar studies and to obtain published results that could be used as benchmarks for the cost-effectiveness results described in our original No-CAUTI study below. Seven relevant studies were published since 2007 [12–18]. All costed interventions that attempted to decrease CAUTI through improved discretionary use of urinary catheters, including their timely removal through electronic reminders and education or teamwork.

The identified studies were set in the USA, the Netherlands and Thailand. All were ‘pre-post’ research designs, except van der Broek et al [18] which used a related ‘step wedge’, for three groups of hospitals. Further, all except van der Broek et al [18], which included 10 hospitals, were conducted at single hospital sites.

None of the studies used a cost-effectiveness approach. Cost buckets fell into three categories: (1) intervention, (2) catheterisations (cost of catheters, nurse administration time), and (3) CAUTI (diagnostics, such as urine tests and antibiotics and length of stay).

van der Broek et al. [18], reported changes in prevalence of indwelling urinary catheterisations by ward (pre and post); Pashnick et al. [15] reported change in catheter utilisation per patient days; and Cho et al. [13] reported mean change in catheter days. Apisarthanark et al. [12] collected informtion on ‘appropriate’ and ‘inappropriate’ catheterisations (as defined by the authors) and reported changes in mean rates of ‘inappropriate catheter days’. Sutherland et al[16], reported CAUTI rates as increasing three years post-intervention, demonstrating a decay in intervention effectiveness.

Only two studies [18, 14] costed their interventions; the first gathering costs through records of labour time maintained by the intervention group; the second, from hospital administrative records and nurse case managers. Van der Broek et al [18] was also the only study to compare pre and post catheterisations costs. It was also the only study to cost comparative catheter incidence between the two groups; and the only study not to cost CAUTI diagnostics or treatments or hospital stay.

The following cost findings are given in 2019 Australian dollars (2019-20 AUD) calculated with exchange rates published by the Reserve Bank of Australia [19] and adjusted for inflation with the Australian Bureau of Statistics (ABS) Consumer Price Index [20]. Over 10 Dutch hospitals, the intervention evaluated by Van der Broek et al [18] cost between $2,103 and $7,736, per hospital. The mean hospital cost was $5,423. In a study that included hospitalisation costs, including length of stay, in their CAUTI cost calculations, Clarke et al [14] found that their bundle of four staggered interventions had an annual estimated cost of $36,436. The authors conducted their intervention in a single hospital, as an ongoing activity, while van der Broek et al [18] delivered their intervention once.

Pashnik et al [15] reported $9,728 per CAUTI incidence, without specifying the exact cost buckets but reported that these costs included diagnostic tests and length of stay. This was an incremental cost calculated by subtracting hospital costs of patients not diagnosed with CAUTI, from the hospital costs of patients diagnosed with CAUTI. It can be considered as the value of a ‘CAUTI prevented’. Apisarthanark et al [12] separately reported ‘hospitalisation costs’ at pre, $658 and post, $277, a saving of $481. This was for a respective reduction in inappropriate catheterisations in pre and post group from 225 (20.4 %) to 144 (11 %).

## Methods

### No-CAUTI Intervention

The No-CAUTI trial set out to reduce indwelling catheterisations among over-night patients in four hospitals. No-CAUTI was a nurse-led multi-faceted intervention with four components:

- Catheter insertion criteria guidelines
- Insertion and maintenance care bundle
- Nurse-led catheter removal protocol
- Clinician competency assessment framework

A standardised generic catheter insertion pack with all catheterisation equipment, documentation stickers, and securing devices, was developed. Clinician and patient education resources, which included a poster with insertion and removal guidelines and patient information sheets were also part of the intervention.

No-CAUTI was conducted between 2017 and 2018 across two local health districts (LHDs) in New South Wales (NSW), Australia, within four acute care hospitals, in each of the respective LHDs. Two hospitals were in metropolitan locations, the others in rural areas. The metropolitan hospitals had bed capacities of 550 and 318 beds, each with an ICU. The rural hospitals had separate capacities for 360 and 260 beds. The first housed a Level 5 ICU, the other had a Level 3 high dependency unit (HDU).

 A staggered implementation was used where the intervention was deployed in both hospitals in one LHD, followed four-months later in the two hospitals in the second LHD. In both LHDs, the intervention required six months for implementation, implementing the above components with a series of strategies.

Education was provided as Train-the-Trainer workshops so that ongoing training could be provided internally at each site into the future, particularly for orientation of new staff. Ward level in-services were then carried out over a 4-week period by those who were trained in workshops to snowball the education process. Champions were identified and established on each ward to act as a resource for clinicians, promote the No CAUTI bundle of care and support implementation. Monthly meetings were held with champions to identify issues related to uptake and find solutions.

Compliance audits were initially attended weekly and then monthly to monitor bundle compliance and uptake, and also to empower champions and clinicians to continue targeted implementation strategies.

Promotional material such as posters, a video and a website were developed and distributed to provide ongoing resources for education, and to continue to raise awareness of the No CAUTI bundle.

Audits for compliance were completed by nurses, nurse educators or midwives on all research wards, weekly for six weeks, and thereafter each month for six months. Intervention implementation strategies are described in further detail elsewhere [9, 11]. Ward-in-services were not costed in this study as they also form part of usual care. Champion meetings were also not costed as they would not be part of a rollout implementation (as advised by author, MG).

### Research Design and Data Collection

This analysis used the research design of the No-CAUTI trial (‘a cluster-controlled pre- and poststudy at a facility level with a phased intervention implementation approach’[11]) which was a pre-post control-intervention, for which the main outcome variable was, catheterisation rate [9, 11].

Unlike studies found in the literature review, this study used a point prevalence approach to collecting patient data. Three point-prevalence surveys of overnight adult patients were conducted across the hospitals. The time points were at: (1) pre-intervention (n = 1630), (2) four-months-post-intervention (n = 1677), and (3) nine months-post-intervention (n = 1551). Each survey took place over two separate days at least 10 days apart to gather adequate numbers.

Data were collected using patient chart audits and extractions from electronic clinical information systems. These data indicated whether each patient during their current visit had: received a urinary catheter in-situ, been tested for urinary tract infection, had received antibiotics for suspected urinary tract infection, or had been diagnosed for CAUTI. The surveys were conducted without opportunity for follow up. More information is available in a related publication [11].

Data was collected for all four hospitals across all overnight adult patients, with the exclusion of ED patients. The time horizon from the pre-intervention point prevalence survey to the 9-months post-intervention survey was 16 months. Further details on the research design and populations appear elsewhere [9, 11].

### Cost Effectiveness Analysis

A cost-effectiveness analysis was undertaken for which the measure of effect was prevalence of indwelling urinary catheterisations. The analysis incorporates costs and catheterisations in ‘per patient’ denominations [21].

The costing perspective is that of the NSW Ministry of Health. Analysis costs included resources for the intervention (education workshops, promotional materials); and offset costs, including procedures and treatments (catheterisations, urine tests, antibiotics) and extra time in hospital associated with CAUTI diagnoses.

Two analyses were conducted to estimate cost effectiveness consistently with the related effectiveness paper [11]. These separately compare ratios of costs per patient to the measured effect (catheterisation rate) for the pre-intervention time point and two post-intervention time points (4-months and 9-months). This approach indicated the consistency of the intervention cost efficiency at two post intervention time points. The two incremental cost-effectiveness ratios (ICERs) are computed as:

$$ \mathrm{ICER}\ 4\hbox{-} \mathrm{months}\ \mathrm{Post}\hbox{-} \mathrm{intervention}=\kern0.5em \mathrm{Costs}\ \left(4\hbox{-} \mathrm{months}\ \mathrm{Post}\hbox{-} \mathrm{Intervention}\right)-\mathrm{Costs}\ \left(\mathrm{Pre}\hbox{-} \mathrm{Intervention}\right)/\mathrm{Catheterisation}\ \mathrm{rate}\ \left(4\hbox{-} \mathrm{months}\ \mathrm{Post}\hbox{-} \mathrm{Intervention}\right)-\mathrm{Catheterisation}\ \mathrm{rate}\ \left(\mathrm{Pre}\hbox{-} \mathrm{Intervention}\right) $$

$$ \mathrm{ICER}\ 9\ \mathrm{months}\ \mathrm{Post}\hbox{-} \mathrm{intervention}=\kern0.5em \mathrm{Costs}\ \left(9\hbox{-} \mathrm{months}\ \mathrm{Post}\hbox{-} \mathrm{Intervention}\right)-\mathrm{Costs}\ \left(\mathrm{Pre}\hbox{-} \mathrm{Intervention}\right)/\mathrm{Catheterisation}\ \mathrm{rate}\ \left(9\hbox{-} \mathrm{months}\ \mathrm{Post}\hbox{-} \mathrm{Intervention}\right)-\mathrm{Catheterisation}\ \mathrm{rate}\ \left(\mathrm{Pre}\hbox{-} \mathrm{Intervention}\right) $$

Compared to the pre-intervention period, the ratios represent the net opportunity cost per unit of change in catheterisations for patients at the respective post-intervention time points [21].

Data was collated and modelled in a Microsoft Excel 2016 [22] workbook. Visual Basic for Applications (VBA) [23] was used to program sensitivity analyses and non-parametric bootstrapping [24.] A schematic of the cost effectiveness model appears in Additional File 1 (1_Model Schematic)*.* As the modelling is undertaken within a period of less than 12 months, no discounting was applied.

### Costing

This section offers further detail on the costs used in the modelling. All costs are given in 2019–2020 prices. Inflation adjustments were made with the Total Health Price Index published by the Australian Institute of Health and Welfare (AIHW) [25]. On-costs (payroll tax, superannuation etc.) are applied to all base labour costs at 21.5 % [26]. Overheads (electricity, building maintenance etc.) are costed at 27.5 % of labour (Personal Communication between RL and John Hunter Hospital Finance Officer, 2019) and applied to all labour activities (base rates). Note that all quoted per hourly rates in the text refer to base rates as published in wage awards.

For modelling and data collection purposes, this study took a ‘micro-costing’ or ‘bottom up’ approach. All relevant individual costs were identified, collected, quantified, valued, tabulated and aggregated to build estimations of total costs [24]. All research costs are excluded, as these are ‘sunk costs’ that will not be replicated in wider implementation of the intervention. All quoted labour costs include on-costs and added overheads.

The nine three-hour workshops were each costed as three hours of labour for a Clinical Nurse Consultant Grade 3 (CNC3) instructor, over the four facilities [27]. Labour costs of all attendees were included [21], valued at the ‘Full-time adult average weekly ordinary time earnings’ for Australia [28] (Additional File 1 (2_Unit Labour Costs)).

 Compliance auditing and point prevalence surveys were costed here as per a No-CAUTI roll-out. As advised by author, MG, audits would be conducted only for wards where indwelling catheters have been administered, comprising some bedside observation of catheterised patients and inspection of their notes. Audit durations across the hospitals were calculated on MG’s estimate that the largest hospital would require approximately two hours. (Additional File 1 (5_Audits_Point_Prev)). For a roll out across the trial hospitals, it is expected that compliance audits would be conducted weekly for the first four weeks, each by a registered nurse (Year 8 or above (RN8)) [27]. Thereafter, one audit would be conducted annually – including at the end of year 1. Accordingly, five compliance audits have been costed for each hospital (Additional File 1 (5_Audits_Point_Prev)). Also, in a roll out, point prevalence surveys would occur once in the pre-intervention period, and then annually. Each would require two nurses at RN8 level. For the current study, two point-prevalence studies have been costed for each hospital. The researchers estimated that the time per hospital would vary by bed numbers with the largest hospital (550 beds) requiring a full eight-hour day. Survey durations for the other hospitals were estimated according to proportionate bed numbers.

In common with other studies, this one included offset costs associated with CAUTI diagnoses and treatments, including urine tests, antibiotics and increased hospital stays due to CAUTI [12, 14, 16, 29–31] Catheterisation costs included an expected 30 min of nurse labour (expected level, Registered Nurse Year 4 (RN4)) [27] to insert and remove catheters (20 min to insert and 10 min to remove). These expected parameters were provided by researcher MG, a qualified and experienced nurse. Urine testing costs were calculated with reference to Australia’s Medical Benefits Schedule (MBS) (items 6933 and 73930) (Table 1), (Additional File 1, (7_Offsets)) [32].

Increased lengths of hospital stay due to CAUTI diagnoses were also costed [12, 14–16]. By their point prevalence nature, the surveys did not provide length of stay data. Hence, estimates for expected extra length of stay associated with CAUTI were obtained from the literature. Yi et al [33] estimated that non-ICU patients with CAUTIs had a median extra 3.6 days of hospital. While not distinguishing CAUTIs from other forms of hospital acquired urinary tract infection (HAUTI), Mitchell et al [1] found a similar estimate, where patients with HAUTIs experienced an expected extra length of stay of 4.0 days (3.1 to 5.0). This study follows Mitchell and assumes an extra 4.0 hospital days for each CAUTI patient.

The cost of each extra day spent in hospital was based on the metric, ‘admitted care average cost per day’, as determined by the Independent Hospitals Pricing Authority and adjusted for inflation to 2019-20 Australian dollars [34, 35], the cost per night calculated at $1971. The total extra cost over four days would hence be, $7884 (Table 1).

Antibiotics were costed at Dispensed Price for Maximum Quantity (DMPQ) rates as published by the Pharmaceutical Benefits Scheme (PBS) web site [36]. Specific antibiotics were identified in the point prevalence data but costed at a calculated ‘average antibiotic cost’ ($19.48) to simplify analysis (Additional File 1, (6_Antibiotics)). Each patient receiving antibiotics was assumed to have been issued a single course.

**Table 1:** Offset Treatment/Procedure Unit Costs

	Cost per Unit ($)
**Catheterisations**	
Nurses Labour: Catheterisation*	$27.46
Catheter Packs ($/pack)	$8.14
**Total**	**$35.60**
**Treatments**	
Urine Specimen Collection/Testing ($/test)	$35.23
Antibiotic prescriptions	$19.48
CAUTI – Four Extra Hospital Days)	$7884.00
*Includes On-Costs and Overhead	

#### Uncertainty Analysis

Calculations of ICERs for both 4-months and 9-months post-intervention surveys were subject to non-parametric bootstrapping [37] over 1,000 iterations and the results plotted on cost effectiveness planes for consideration of point estimate robustness [37].

### Sensitivity Scenarios

Five sensitivity scenarios were designed to recalculate the 4- and 9- months post-intervention ICERs with adjustments to parameters and costs with uncertainty and for latent CAUTIs not detected in the point prevalence data [21]. The scenarios are:

- S1 Intervention Costs: 10 % Increase
- S2 Intervention Costs: 10 % Decrease
- S3 Post Intervention Catheterisation Rates:10 % Increase
- S4 Post Intervention Catheterisation Rates: 10 % Decrease
- S5 Urine Tests: 9 % of are treated as latent CAUTIs.

Consistent with Skelton et al [38], Scenario 5 identifies 9 % of catheterised patients who also received a urine culture test as ‘latent CAUTI cases’ (Additional File 1 (8_Scenario 5)). These patients are costed for four extra days of hospital stay [1].

### Research Ethics and Trial Registration

The study was granted ethical approval by the Hunter New England Human Research Ethics Committee (Ref no. 16/02/17/4.09) and the University of Newcastle Human Research Ethics Committee (Ref no. H-2016-0123). It was also registered retrospectively with the Australian New Zealand Clinical Trials Registry (ACTRN12617000090314 (17 January 2017, (retrospectively)). The first hospital enrolment was at 15/11/2016; while the last enrolment was at 8/12/2016 [8, 9]. The study adhered to the Consolidated Health Economic Evaluation Reporting Standards (CHEERS) and Consolidated Standards of Reporting Trials (STROBE) guidelines (Additional Files 2 and 3). The original effectiveness study [11] was performed in conformance with the StaRI checklist.

## Results

### Patient Characteristics

There were no statistically significant differences among the survey populations for gender, age, and intensive care unit status. Respective mean ages were 69, 70 and 69 years. A more detailed description of the populations appears elsewhere [11].

### Costing Results

A total of 81 persons attended the nine education sessions, each of three hours. Workshop costs for the entire intervention were $18,837 (Additional File 1 (3_Workshops)). Costs for promotional items (posters, badges, and flyers) totalled $7145 (Additional File 1 (4_Promotional Items)) (Table 2).

**Table 2:** Intervention Costs

	($)
No-CAUTI Training Workshops	$18,837
Compliance Audits/Point Prevalence Surveys	$6828
Promotions (Posters/Badges etc.)	$7145
**Total**	**$32,810**

The total compliance and point prevalence auditing costs – as expected for an intervention roll out, were estimated at $6828 (Additional File 1 (5_Audits_Point_Prev)) (Table 2).

Total costs for the intervention were $32,810 (Table 2). As the analysis is limited to ‘in-trial’ consideration, the cost of the intervention – which was completed once to treat all inpatients admitted afterward – will be allocated evenly across inpatients in both post-intervention point prevalence surveys. This effectively standardises the intervention cost, as would be appropriate in planning an intervention for patients across time, leaving average cost per patient the same for both surveys. Hence, the average cost per patient was $10.16 (4-months post-intervention (n) = 1677; plus 9-months post-intervention (n) = 1551, total patients = 3228; $32,810/3228=$10.16 per patient).

### Outcomes

For the three-point prevalence surveys – pre-intervention, 4- and 9- months intervention - total patients with catheters *in situ*, were respectively, 195 (12.0 %(10.4 − 13.5 %)), 166 (9.9 %(8.5 − 11.3 %)) and 158, (10.2 % (8.7 − 11.7 %)). Note a decrease between pre-intervention and 4-months intervention; and a subsequent increase at 9-months intervention.

As noted elsewhere [11], these results show a statistically non-significant trend (*p* = .1) towards reduction of catheter prevalence overall across the three timepoints. However, given catheterisation rates pre-intervention (12 %) were considerably lower than rates reported in the literature (15 to 25 %) [39, 40], a 2 % overall reduction in catheter prevalence to 10 % post-intervention is clinically significant. Further, reductions in catheter prevalence were more marked in hospitals that started with a higher baseline, evidenced by a statistically significant difference in urinary catheterisations at one rural hospital from pre-intervention to 9-months post-intervention (16–8 %, *p* < .01). Statistically significant decreases also occurred between pre-intervention to 4-months post-intervention for medical wards (*p* = .02); pre-intervention to 4-months post-intervention for critical care wards (*p* = .05); and pre-intervention and 9-months post-intervention for female patients (*p* = .015).

Between pre-intervention and 9-months post-intervention, catheterisations for urinary retention (i.e. non-avoidable catheterisations) increased from 29 to 41 %; while postsurgical catheterisations (potentially more avoidable and shorter term), decreased from 29 to 21 % [11]. This shows the intervention as clinically effective among avoidable catheterisations. There was in fact, a significant reduction in the short-term catheter group (≤ 3 days) *(p =* .023*).* The intervention had a clear impact on short-term catheter prevalence, and this was maintained at 9-months post-intervention.

Urine testing numbers were (% of catheterised patients) 85 (43.6 %), 57 (34.3 %) and 81 (51.3 %). Among catheterised patients, the respective numbers receiving antibiotics for ‘suspected urinary tract infections’ were 15 (7.7 %), 10 (6.0 %) and 14 (8.9 %) (Table 3).

**Table 3:** CAUTI trial: outcomes, costs, incremental cost effectiveness. Pre intervention, post four months & nine months post intervention

			Pre-Int	Post 4-Months	Post 9-Months
**1. Overnight Hospitalisations (n)**	a		1,630	1,677	1,551
**2. Catheterisations**					
**Observed Catheterisations (n)**	b		195	166	158
**Catheterisations rates (%)**	c	b/a	**12.0%**	**9.9%**	**10.2%**
**3. CAUTIs (Among catheterised patients)**					
**CAUTIs (n)**	d		**1**	**0**	**0**
**CAUTI rates (% of catheterised)**	e	d/b	**0.51%**	**0.00%**	**0.00%**
**Urine tests (n)**	f		**85**	**57**	**81**
**Urine tests (% of catheterised)**	g	f/b	**43.6%**	**34.3%**	**51.3%**
**Antibiotic Administrations (n)**	h		**15**	**10**	**14**
**Antibiotic Administrations (% of catheterised)**	i	h/b	**7.7%**	**6.0%**	**8.9%**
**4. Aggregate Costs**					
**Intervention**	j		**$0**	**$17,045**	**$15,765**
**Offsets**					
**Catheterisations**	k		$6,941	$5,909	$5,624
**CAUTI Related (Urine Tests and Antibiotics)**	l		$3,287	$2,203	$3,126
**Extra LOS due to CAUTI**	m		$7,884	$0	$0
**Total Offsets**	n		**$18,112**	**$8,112**	**$8,751**
**Total Aggregate Costs**	o	j+n	**$18,112**	**$25,158**	**$24,516**
**5. Average Costs (per hospitalisation)**					
**Intervention**	p	j/a	**$0.00**	**$10.16**	**$10.16**
**Offsets**					
**Catheterisations**	q	k/a	$4.26	$3.52	$3.63
**CAUTI Related (Urine Tests and Antibiotics)**	r	l/a	$2.02	$1.31	$2.02
**Extra LOS due to CAUTI**	s	m/a	$4.84	$0.00	$0.00
**Total Offsets**	t		**$11.11**	**$4.84**	**$5.64**
**Total Average Costs (per hospitalisation)**	u	p+t	**$11.11**	**$15.00**	**$15.81**
**6. Incremental Costs on Pre-Int (per hospitalisation)**					
**Incremental Costs**					
**Intervention**	v	increment p		$10.16	$10.16
**Catheterisations**	w	increment q		-$0.73	-$0.63
**CAUTI Related (Urine Tests and Antibiotics)**	x	increment r		-$0.70	$0.00
**Extra LOS due to CAUTI**	y	increment s		-$4.84	-$4.84
**Total Incremental Costs**	**z**			**$3.89**	**$4.69**
**7. Decreases in Catheterisation rates**	aa	**increment c**		**2.1%**	**1.8%**
**8. Incremental Cost Effectiveness Ratio (ICER)**	ab	**z/aa**		**$188**	**$264**

Rounding errors apply

It is noted that the trial observed only one diagnosed case of CAUTI, which appeared in the pre-intervention survey [11]. However, this finding does not undermine any subsequent analysis, as (1) the primary outcome for the trial was, catheterisation rates; (2) the point prevalence survey approach has a chance of missing latent CAUTIs, which may have been later diagnosed among patients receiving antibiotics for urinary tract infection symptoms. The possible economic consequences of possible latent CAUTIs among the study populations will be explored in Sensitivity Scenario 5.

### Cost-effectiveness Analysis

As previously noted, the trend in catheterisation rates - the outcome variable - was found to be statistically non-significant. The differences in catheterisation rates between pre-intervention and 4-months intervention; and pre-intervention and 9-months post intervention; are accepted as being due to random variation. Cost effectiveness analyses have been conducted in the light of findings of clinical effectiveness, discussed earlier, which occurred at both post intervention points. Consideration of cost effectiveness at 4- and 9- months intervention also allows consideration of potential resource costs of maintaining the intervention.

Table 3 contains a summary and breakdown of the point cost-effectiveness findings. The incremental cost effectiveness ratios (ICERs) - at the very bottom of table - show that at 4-months post-intervention, the cost of one less catheter insertion as compared to pre-intervention was $188. At 9-months post-intervention, the estimated cost of one less catheter insertion was $264.

### Uncertainty Analysis

**Fig. 1:**
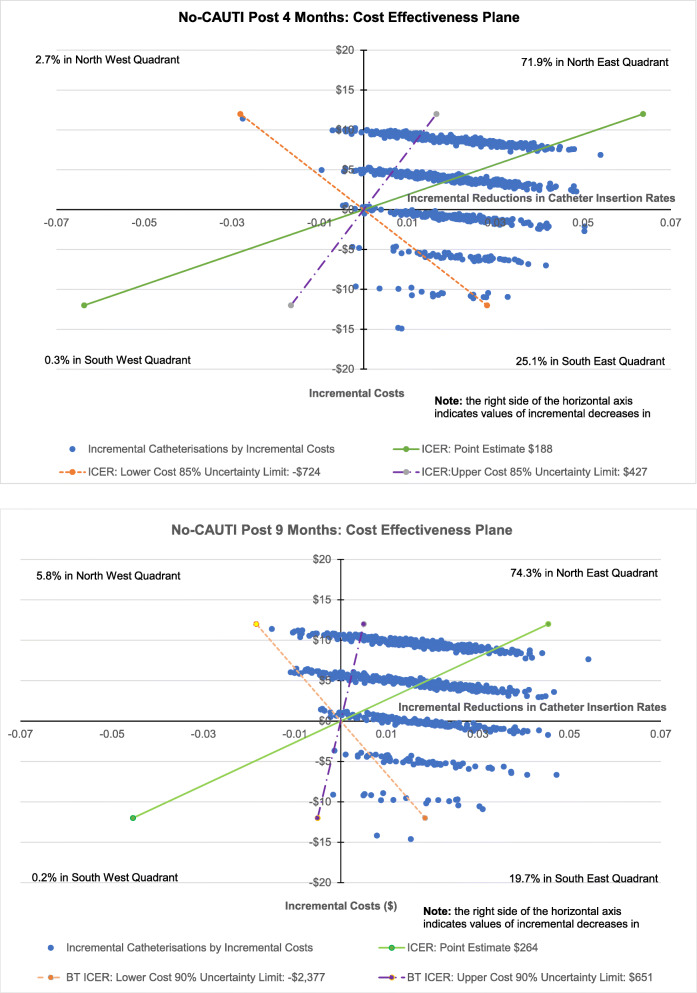
Cost-effectiveness Planes Base Case: Pre vs. Post-Intervention (a) 4-months (b) 9-months.

To consider uncertainty of point ICERs, data for both 4- and 9-months post-intervention was subject to a non-parametric boot strapping with 1,000 iterations [37]. As stated above, the 4-months-post-intervention ICER, indicated a cost of $188 of resources for a unit decrease in catheterisations. Among bootstrap results, 71.9 % fell in the north-east quadrant indicating an intervention associated reduction in catheterisations with additional costs (resources used). Also, 25.1 % appeared in the south east quadrant, showing decreases in catheterisations but with a negative cost (saving of resources) (Fig. 1).

Also as stated above, the 9-months post-intervention CEA had a point estimate ICER of $264. Among bootstrap results, 74.3 % fell in the north-east quadrant and 19.7 % in the south east quadrant (Fig. 1).

### Sensitivity Scenarios

The results for sensitivity scenarios are presented in Table 4. Scenarios 1 and 2 consider 10 % upward and downward adjustments in the intervention costs, showing respective uniform shifts in 4 months and 9 months ICERs of 26 and 22 %. On the expectation that 10 % is a reasonable variation in intervention cost, this establishes intervals around the base case ICERs at 4-months ($139 - $238) and 9-months post intervention ($207 - $322).

Scenarios 3 and 4 allow for 10 % changes in the outcome variable – catheterisation rate – at the post intervention points. Note that for scenarios 3 and 4, the modelling adjusts post-intervention numbers of urine tests and antibiotic administrations, keeping their incidence in relation to catheterisations the same as for base case and adjusting associated costs. For Scenario 3, the 4- and 9- month post intervention ICERs respectively increase to $418 and $711. Also, for Scenario 4, the respective 4-month and 9-month adjusted ICERs are $110 and $146. These shifts are more dramatic as the catheterisation rate is the denominator of the ICER formula.

Scenario 5 recognised 9 % of catheterised patients receiving urine culture tests as latent CAUTI cases [38]. As observed at pre-intervention, post 4 months and post 9 months, numbers of catheterised patients who received urine cultures were (percentages of catheterised patients in brackets) 85 (43.6 %), 57 (34.3 %) and 81 (51.3 %). For Scenario 5, numbers of latent CAUTI cases at pre-intervention, and post-intervention 4-months and 9-months are: 8, 5 and 7.

Between pre intervention and 4 months post-intervention there is a cost saving of $313 on every reduced catheterisation, as forecast CAUTI numbers declined from 8 to 5. However, the difference in CAUTIs between pre-intervention and post-intervention 9 months is only one (8 versus 7), and the ICER rebounds to $361.

**Table 4:** Sensitivity Scenarios

	Post 4 Months		Post 9months	
	**ICER ($)**	**∆ Base Case (%)**	**ICER ($)**	**∆ Base Case (%)**
**Base Case**	**$188**		**$264**	
S1 Intervention Costs: 10 % Increase	$238	26.%	$322	22 %
S2 Intervention Costs: 10 % Decrease	$139	-26.%	$207	-22 %
S3 Post Intervention Catheterisation Rates: 10 % Increase	$418	122 %	$711	170 %
S4 Post Intervention Catheterisation Rates: 10 % Decrease	$110	-42 %	$146	-45 %
S5 Urine Cultures 9 %: Latent CAUTIs	-$313	-266 %	$361	37 %

## Discussion

To the best of the authors’ knowledge, this is the first cost-effectiveness analysis of a nurse-led catheter reduction program in a hospital setting. As noted above, the general changes in the main outcome variable - indwelling urinary catheterisation rate - were statistically nonsignificant for the trend between pre-intervention and the post-intervention stages. However, significant reductions and examples of clinical effectiveness were noted in: one hospital for the pre to 9-months post-intervention interval; overall in the placement of short term catheterisations; and for female patients. [11]. It is also noted that an increase in the catheterisation rate was detected at 9-months post intervention suggesting a decay in the trial-effect, although statistically this was found to be a non-significant change on the pre- period catheterisation rate.

Incremental cost-effectiveness ratios (ICERs) respectively at 4- and 9-months post-intervention points were, $188 and $264. For non-parametric boot strapping on both ICERS approximately 72–75 % of iterations showed decreases in incremental catheterisations with positive costs (Fig. 1).

Sensitivity scenarios (Table 4) showed that ICERs are most sensitive to changes in post-intervention catheterisation rates. A critical task of implementing No-CAUTI would therefore be to maintain catheterisation rate decreases.

### Policy Implications

Translation of evidence into practice is a key priority area for health jurisdictions in NSW and the NSW Ministry of Health; as is eliminating low-value-high cost care. The No-CAUTI intervention may be cost-effective in acute care health services, where saving healthcare dollars is a key priority.

The primary management issue for a No-CAUTI intervention is in maintaining catheterisation rate reductions after intervention. The No-CAUTI intervention requires maintenance of best practice and continued focus on avoidance of unnecessary catheterisations. Such a maintenance will bring increases in hospital efficiency, and better use of resources. For this reason, ongoing monitoring and evaluation needs to be built into the No-CAUTI implementation model and perhaps performed more regularly.

This cost-effectiveness study does not directly estimate the impacts of a roll out of the No- CAUTI intervention on health budgets or give information on its affordability [41].

## Limitations

The major limitation of this study is in the data collection method, point prevalence surveying. The intervention effect – catheterisation rates – would be more validly measured cumulatively across flows of patients, especially given that catheter duration is a key consideration when considering the risk of CAUTI. However, as the analysis is based on point prevalence surveys, there was no measurement of cumulative quantities of catheterisations over time. Also, any observations with extreme values will bring unknown bias to the study. However, point prevalence surveys are often the only practical choice. This is due to difficulties in: collecting data over continuous periods due to poor documentation of urinary catheter insertions; gaining access to patient records; or in acquiring funding for larger surveys. The restriction of the study to only four hospitals is also a limitation. Another is the lack of more data points which would have allowed for more thorough statistical analysis of the outcome and uncertainty analysis. A random robust sample of hospitals across NSW would have allowed wider observation of cost variations characteristic of hospitals operating under a broader range of facilities and patient catchments. Last, an extended analysis could also add a patient perspective including private health insurance coverage.

## Summary and Conclusion

The research findings demonstrate how the No-CAUTI intervention may reduce catheterisations and have potential impact in the reduction of resource use associated with CAUTI. Incremental CEAs show that at 4-months post-intervention, the costs of one less urinary catheter insertion as compared to prevalence at pre-intervention, was $188. At 9-months post-intervention, the cost of generating a reduction of one insertion was higher at $264. The study has limitations related to the data gathering approach of point prevalence surveys. The analysis shows that given clinical significance, the intervention has a high possibility of incremental cost efficiency at the observed levels.

## Supplementary Information

**Additional file 1:** Supporting calculations referred to throughout the text 

**Additional file 2:** Populated CHEERS Checklist 

**Additional file 3:** Populated Strobe Checklist 

## Data Availability

The data was sourced from the No-CAUTI trial (registration and ethics approvals details above).[11] The data that supports the findings of this study are available from the authors, but restrictions apply to the availability of these data, which were used under license for the current study, and so are not publicly available. Data are however available from the authors upon reasonable request and with permission of co-author, Michelle Giles.

## Abbreviations

CAUTI: catheter-associated urinary tract infection; BIA:Budget Impact Analysis; NSW:New South Wales; ICU:intensive care unit; LHD:Local Health District; CNC3:Clinical Nurse Consultant Grade 3, RN8:Registered Nurse Year 8th year and thereafter; Administrative and Clerical Officer Grade 6 Service Thereafter Y1 (ACO6).
